# Translating the fabrication of protein-loaded poly(lactic-co-glycolic acid) nanoparticles from bench to scale-independent production using microfluidics

**DOI:** 10.1007/s13346-019-00699-y

**Published:** 2020-01-09

**Authors:** Carla B. Roces, Dennis Christensen, Yvonne Perrie

**Affiliations:** 1 Strathclyde Institute of Pharmacy and Biomedical Sciences, University of Strathclyde, 161 Cathedral St, Glasgow, G4 0RE Scotland; 2 Center for Vaccine Research, Statens Serum Institut, Copenhagen, Denmark

**Keywords:** Microfluidics, PLGA, Nanoparticles, Proteins, Adjuvants, Polymers

## Abstract

In the formulation of nanoparticles, poly(lactic-co-glycolic acid) (PLGA) is commonly employed due to its Food and Drug Administration and European Medicines Agency approval for human use, its ability to encapsulate a variety of moieties, its biocompatibility and biodegradability and its ability to offer a range of controlled release profiles. Common methods for the production of PLGA particles often adopt harsh solvents, surfactants/stabilisers and in general are multi-step and time-consuming processes. This limits the translation of these drug delivery systems from bench to bedside. To address this, we have applied microfluidic processes to develop a scale-independent platform for the manufacture, purification and monitoring of nanoparticles. Thereby, the influence of various microfluidic parameters on the physicochemical characteristics of the empty and the protein-loaded PLGA particles was evaluated in combination with the copolymer employed (PLGA 85:15, 75:25 or 50:50) and the type of protein loaded. Using this rapid production process, emulsifying/stabilising agents (such as polyvinyl alcohol) are not required. We also incorporate in-line purification systems and at-line particle size monitoring. Our results demonstrate the microfluidic control parameters that can be adopted to control particle size and the impact of PLGA copolymer type on the characteristics of the produced particles. With these nanoparticles, protein encapsulation efficiency varies from 8 to 50% and is controlled by the copolymer of choice and the production parameters employed; higher flow rates, combined with medium flow rate ratios (3:1), should be adopted to promote higher protein loading (% wt/wt). In conclusion, herein, we outline the process controls for the fabrication of PLGA polymeric nanoparticles incorporating proteins in a rapid and scalable manufacturing process.

**Figure:**
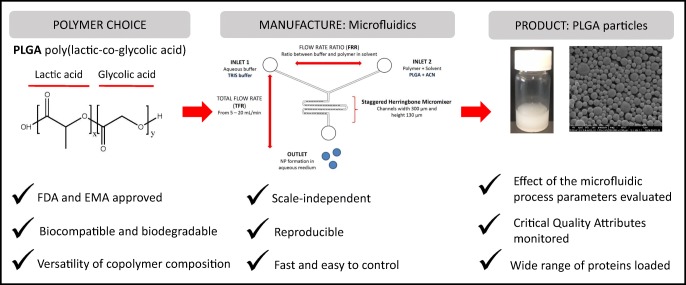
Scale-independent production of polymer nanoparticles

Scale-independent production of polymer nanoparticles

## Introduction

Poly(lactic-co-glycolic acid) (PLGA) is a polyester, which degrades by hydrolysis of the ester connections into its monomer components lactic (PLA) and glycolic acid (PGA) and is commonly used in the production of nanoparticles that are investigated for the delivery of drugs and vaccine antigens. These nanoparticles can be manufactured through a wide range of methods, and the physicochemical properties (size, morphology, surface charge) can be easily manipulated in order to promote appropriate biological activity e.g. [1–3]. When selecting which PLGA to be used within nanoparticles, the monomer molar ratio, the average molecular weight, the degree of crystallinity, the size and shape of monomer are all important. The main PLGA copolymers used in research are 50:50, 65:35, 75:25 and 85:15 (PLA:PGA ratio). Varying the percentage of PLA or PGA present in the polymer composition influences the hydrophilicity of the polymer and thus the polymer degradation rate and release rate of the entrapped moiety [4–6]. Therefore, PLGA copolymers with higher PGA content show accelerated degradation when compared to PLGA copolymers with higher PLA content.

When considering the manufacturing process adopted for the production of nanoparticles, both the particle attributes and the complexity of the process are considerations. With many of the current manufacturing methods, scale up is difficult and requires large amounts of solvents. In contrast, microfluidics manipulates the mixing of liquid flows in microsized channels and allows the formation of size-controlled nanoparticles [7]. Advantages of using microfluidics include the opportunity for continuous operation, easy control, high efficiency and low cost, making it an attractive alternative compared to other traditional manufacturing methods [7]. Microfluidics also offers the potential for scale-independent manufacture, which offers additional economic advantages including tailored production scales and smaller facility footprint, which converts into reduce cost of goods. These combined advantages allow for rapid translation from bench to bedside for new polymeric nanoparticle formulations, thereby de-risking their adopting as nanomedicines.

In our laboratories, we have been working with the staggered herringbone micromixer (SHM) in combination with the Nanoassemblr® from Precision Nanosystems Inc. (Vancouver, Canada) for the production of liposomes [8, 9]. Previous studies have demonstrated the formulation of polymeric nanoparticles using microfluidics to incorporate small drugs molecules (e.g. [10, 11]) or different types or RNA (e.g. [12]). However, knowledge of the application of microfluidics to produce protein-loaded nanoparticles is lacking. Furthermore, to advance the progress of microfluidic production, we have also incorporated in-line purification methods and at-line particle size monitoring as part of a continuous manufacturing process. Therefore, the aim of this study was to investigate, develop and define a microfluidic process for the production of protein-loaded PLGA nanoparticles that incorporate purification and process monitoring in a rapid and scalable manner.

## Materials and methods

### Materials

For the preparation of the delivery systems, polymers PLGA 85:15 (Mw: 50,000-75,000), 75:25 (Mw: 66,000-107,000) and 50:50 (Mw: 30,000-60,000) from Sigma-Aldrich were used. Sodium hydroxide (NaOH), polyvinyl alcohol (PVA Mw: 31,000), ovalbumin (OVA) and bovine serum albumin (BSA) were purchased from Sigma-Aldrich Company Ltd., Poole, UK. The tuberculosis vaccine candidate ‘Hybrid 56’ (H56) was donated by Statens Serum Institut (SSI), Copenhagen, Denmark. 2-Amino-2-(hydroxymethyl)-1,3-propanediol (Tris) was obtained from ICN Biomedicals Inc. (Aurora, OH, USA) and prepared at a 10 mM concentration and pH 7.4 unless otherwise stated. Acetonitrile (ACN), trifluoroacetic acid (TFA) and all other reagents were of analytical grade and purchased from commercial suppliers.

### Manufacturing of poly(lactic-co-glycolic acid) nanoparticles using microfluidics

PLGA nanoparticles were manufactured by the microfluidics method using the Nanoassemblr® Benchtop (Precision Nanosystems Inc., Vancouver, Canada). Briefly, polymer (either PLGA 85:15, 75:25 or 50:50) was dissolved in ACN at a concentration of 10 mg/mL (1% w/v). For the production of empty nanoparticles, Tris buffer was used as aqueous phase, whereas for protein-loaded nanoparticles, protein (OVA, BSA or H56) was loaded in the aqueous phase at the desired concentration (0.2, 0.5 or 1 mg/mL). The addition of PVA at different concentrations (0, 0.5, 1 and 2% w/v) into the aqueous phase during the production of PLGA nanoparticles was also tested. In order to evaluate the impact of the different production parameters, total flow rates (TFRs) 5, 10 and 15 mL/min and flow rate ratios (FRR) 1:1, 3:1 and 5:1 were selected. Encapsulation efficiencies (EE%) were calculated as a percentage of the initial protein concentration × the dilution factor from each FRR. Loading capacity (% wt/wt) was calculated as percentage of the mass of protein loaded into the nanoparticles divided by the mass of the whole nanoparticle.

### Characterisation of the PLGA particle size, zeta potential and morphology

The particle size and zeta potential of the polymer nanoparticles were determined by dynamic light scattering (DLS) using a Malvern nano ZS (Malvern PANalytical, Worcestershire, UK). Three measurements at 25 °C were conducted on the samples, which were previously diluted in filtered Tris buffer (10 mM, pH 7.4) to achieve the optimal particle concentration (0.25 mg/mL) with the optimum attenuator number (att. 6–7). To prove the applicability of the microfluidics method for continuous manufacturing, an AT-line Malvern sizer was used to measure the PLGA nanoparticles. The system was run at the following: 0.5 mL/min sample flow rate, 10 mL/min diluent flow rate and 90 s delay time between measurements. To consider morphology, scanning electron microscopy (SEM) was used to visualise the polymer nanoparticles. These were fixed and air dried onto a metal stub and then coated with gold and observed under the microscope. This procedure was carried out by David McCarthy from DMmicroscopy. Images were taken on a FEI Quanta FEG, Eindhoven, the Netherlands. The voltage used is shown at the foot of each image, usually 5 or 8 KV.

### Purification of the PLGA nanoparticles: solvent removal and non-encapsulated antigen

For the removal of the solvent used for the dissolution of the polymer and preparation of nanoparticles, samples were in a dialysis tubing (Mw = 12,000–14,000 Da, Sigma-Aldrich Company Ltd., Poole, UK) against Tris buffer for an hour. PLGA nanoparticles were then purified via tangential flow filtration (KR2*i* TFF System®, SpectrumLabs, USA) at 27 mL/min and 12 diafiltration volumes in order to remove unbound protein. The TFF column used for this purpose was a 750-kDa modified polyethersulfone (mPES) column. Dialysis was carried out before TFF due to the incompatibility of the column with the ACN used for the preparation of the nanoparticles.

### Quantification of protein loading and polymer recovery

Quantification of the protein loading within the PLGA nanoparticles was performed by reverse phase HPLC (RP-HPLC) using a UV detector. Jupiter 5 μ C18(2) column (Phenomenex) pore size 300 Å was used as stationary phase. For the preparation of the samples, proteins and/or protein-loaded nanoparticles were dissolved in 0.1 M NaOH/Tris buffer (1:1 v/v) and heated up for approx. 1.5–2 h in a water bath at 35 °C. A gradient elution method containing 90% H_2_O, 10% ACN and 0.1% TFA in one phase (mobile phase A) and 70% acetonitrile, 30% H_2_O and 0.1% TFA in the other phase (mobile phase B) was followed [13, 14]. Injection volume was 50 μL, flow rate 1 mL/min, UV wavelength 210 nm, column temperature either 25 °C (OVA and BSA) or 60 °C (H56), and the total duration of the run was 20 min.

Quantification of the polymer recovery was performed by HPLC using an ELSD detector based on the method developed by Riehl et al. [15]. Luna 5 μ C18(2) column (Phenomenex) pore size of 100 Å was used. HPLC-ELSD settings were kept constant as follows: 30 μL injection volume in a partial loopfill injection mode, 100 μL loop volume and 15 μL tubing volume. Column temperature was maintained at 35 °C, whereas the ELSD temperature was set at 52 °C in all the runs. Nitrogen was used as a carrier gas at 3.5 bar inlet pressure. Clarity DataApex version 4.0.3.876 was used for data analysis.

### Statistical analysis

One-way ANOVA and two-way ANOVA followed by Tukey’s multiple comparison test were used for the data analysis. All the experiments were carried out at least in triplicate unless otherwise stated. Results are the mean of at least 3 measurements ± standard deviation (SD) which is plotted as error bars.

## Results and discussion

In the development of nanoparticles, the size and size distribution or uniformity, i.e. polydispersity index (PDI), are essential characteristics. Low PDIs are difficult to obtain in some formulations depending on the nature of the compounds used and the manufacturing techniques applied. However, it is necessary to have a narrow range of particle sizes within the samples since a broad range may have different pharmacokinetic profiles; affect cell uptake; and in the role of adjuvants, it can influence their immunogenic properties [16–19]. Similarly the zeta potential can give insights into the surface charge of the particles which can impact on their biological activity [20]. Therefore, our initial studies focused on the effect of microfluidic parameters in combination with PLGA copolymer ratio (50:50; 75:25; 85:15) on the size, PDI and zeta potential of our PLGA particles.

### Microfluidics manufacturing of PLGA nanoparticles: The effect of process parameters

**Fig. 1:**
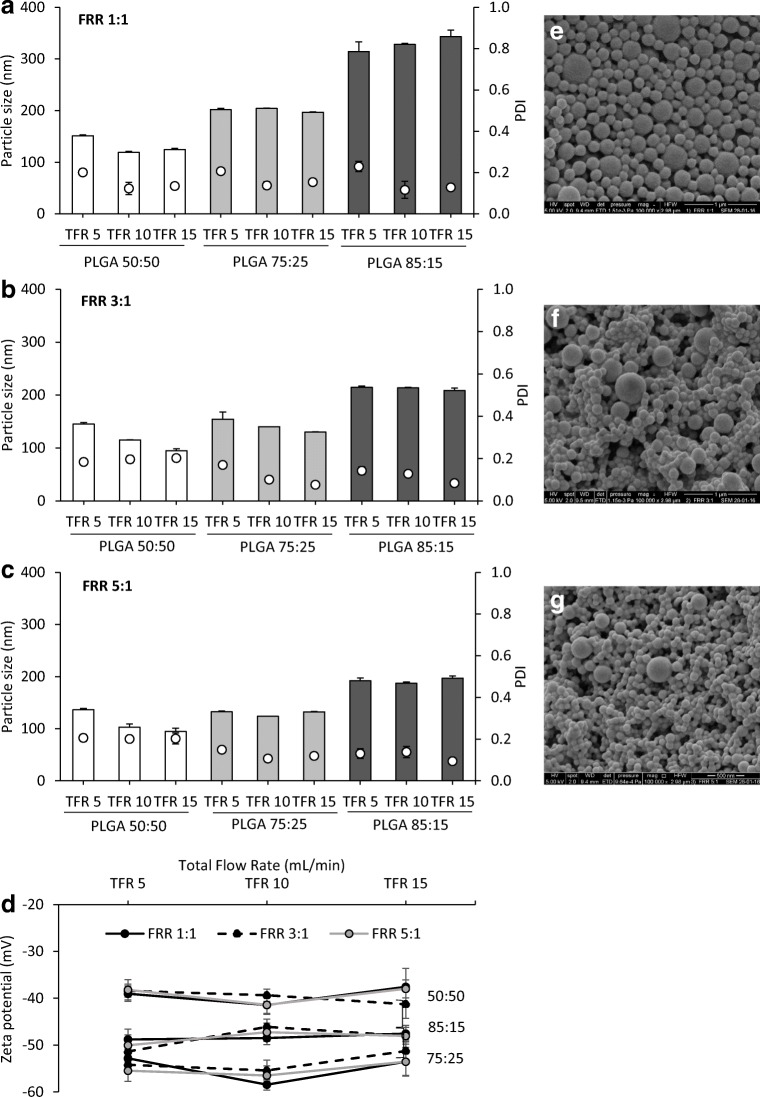
Optimisation of the process parameters for the production of polymeric nanoparticles using microfluidics. Polymeric nanoparticles were prepared by microfluidics, and their physicochemical characteristics (particle size (bars) and PDI (dots)) at **a** FRR 1:1, **b** FRR 3:1 and **c** FRR 5:1 were measured. The effect of FRR on **d** zeta potential was also measured. Results represent the mean ± SD of at least three independent batches. SEM micrographs of **e** PLGA 75:25 nanoparticles FRR 1:1, **f** PLGA 75:25 nanoparticles FRR 3:1 and **g** PLGA 75:25 nanoparticles FRR 5:1 at 10 mL/min TFR

To get an overview of the impact that microfluidic parameters have on the mean particle size, PDI and zeta potential of the PLGA nanoparticles, the TFR and the FRR were varied and assessed (Fig. 1). TFR was set at 3 different speeds: 5, 10 and 15 mL/min and, for each speed, three aqueous:organic phase (buffer:ACN) ratios (FRR) were used: 1:1, 3:1 and 5:1. The initial PLGA concentration in ACN was 10 mg/mL (1% w/v) for all the formulations prepared. Higher (12.5 mg/mL) and lower (7.5 mg/mL) concentrations were also tested and the results did not differ significantly from each other (data not shown), and thus, the concentration was fixed at 10 mg/mL for all the studies carried out.

PLGA nanoparticles produced using the copolymer 50:50 showed the smallest particle sizes among the three PLGA copolymers studied, ranging from 94 to 150 nm, with the particle size reducing as the production speed increased from 5 to 15 mL/min (Fig. 1). However, for copolymers 75:25 and 85:15, there was no notable difference in particle size across the production speeds tested at each of the three FRRs tested (FRR 1:1, 3:1 and 5:1; Fig. 1a, b and c respectively). In contrast, varying the FRR was shown to have a significant (*p* < 0.05) impact on the colloid size with particles decreasing in size for all three copolymers when increasing the aqueous to organic phase ratio from 1:1 to 5:1 (Fig. 1a, b and c, respectively). This trend was observed with all three polymer ratios tested and became more notable with increasing polymer ratio (Fig. 1). In general, across the formulations, the PDI values were ≤ 0.2, and samples had similar morphology when viewed via SEM (Fig. 1e–g). Regarding zeta potential, all three copolymers produced were highly anionic (Fig. 1d). No significant differences were observed between the different microfluidic parameters applied. In general, PLGA 50:50 produced particles with ~ − 40 mV at all FRRs and TFRs tested. Particles prepared using PLGA 85:15 produced particles with ~ − 50 mV, and PLGA 75:25 produced the most anionic particles, with a zeta potential of approximately ~ − 60 mV.

Based on these studies, it can be seen that in terms of size, both TFR and FRR can be considered as critical process parameters, most notably for PLGA 50:50 formulations. However, in the case of the more hydrophobic polymers, the TFR does not have a notable impact. Neither the TFR nor the FRR influence the zeta potential as would be expected given this is dictated by the polymer used. The negative zeta potential is attributed to PLGA preparations due to carboxyl groups present in their structure. Shabir et al. hypothesised that the particle size of the PLGA nanoparticles could influence on the zeta potential due to the reduction of COO^−^ groups available on the surface of the smaller particles and, subsequently, increasing the zeta potential [21]. Chiesa et al. also showed the same trend on PLGA nanoparticles produced using this micromixer system [10]. It has been proposed that FRR impacts on the nanoparticle formation due to the increase in the polarity within the microfluidics cartridge and therefore the different solvent phase concentration [8, 22]. However, it is important to note that the intrinsic factors of the PLGA copolymer also dictate the physicochemical characteristics of the resulting nanoparticles with higher PLA content increasing the particle size irrespective of the process parameters employed. This might a combination of the higher hydrophobicity of the polymer and the manufacturing method, since the polarity during nanoparticle formation is reduced and, thus, larger particles are produced.

### The effect of the addition of a stabiliser into the PLGA nanoparticles

**Fig. 2:**
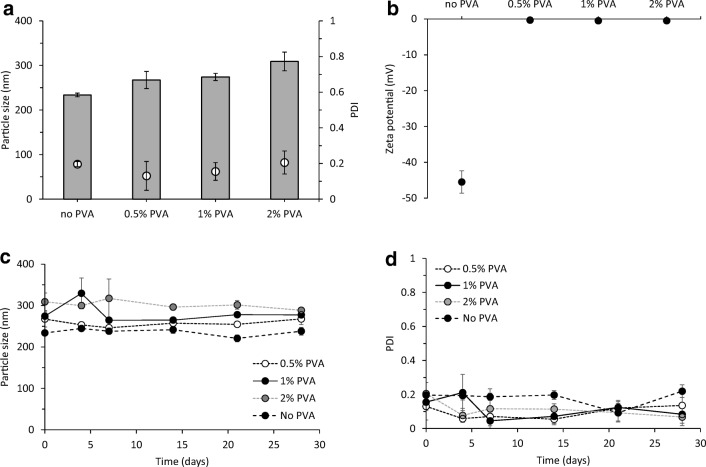
The role of PVA in polymeric nanoparticles prepared by microfluidics. The effect of incorporating different concentrations of PVA (0.5%, 1% and 2% w/v) as stabiliser in the H56 loaded PLGA 75:25 nanoparticles formulated using microfluidics TFR 10 mL/min and FRR 1:1 was investigated in terms of **a** particle size (grey bars) and PDI (white dots) and **b** zeta potential. A stability study of the particles at 4 °C in Tris 10 mM (pH 7.4) was carried out for a month: **c** particle size and **d** size distribution of H56 loaded PLGA 75:25. Results represent mean ± SD of triplicate measurements

The effect of incorporating surfactants/stabilisers into the PLGA nanoparticles formulated using microfluidics was also investigated. PVA is a non-ionic surfactant, which is one of the most common stabilisers used during the emulsification method for the preparation of PLGA particles. PVA favours the emulsification process and avoids the aggregation of PLGA droplets [23]. For this reason, different amounts of PVA were incorporated into the formulations to consider if this enhanced particle formation and/or stability [24–27]. Due to the high hydrophilicity of this polymer, PVA was added into the aqueous phase (Tris buffer pH 7.4, 10 mM) and the stability (at 4 °C) of the particles in terms of size was measured over time (Fig. 2).

Incorporation of PVA produced larger particle sizes, increasing the particle size from approximately 230 to 310 nm with the addition of 2% PVA but did not significantly influence the PDIs, with all formulations having a 0.2 or below, which is representative of a homogeneous particle size population (Fig. 2a). The zeta potential of the PLGA nanoparticles changed from highly anionic to neutral upon addition of PVA in the formulations (Fig. 2b). However, irrespective of the PVA content, all four formulations (no PVA, 0.5% PVA, 1% PVA and 2% PVA) were stable over the period of the study, demonstrating that there is no need for the incorporation of the emulsifier when polymeric nanoparticles are produced via microfluidics (Fig. 2c, d).

The increase in particle size with the addition of PVA has been reported in other studies. For example, Chiesa et al. added the same concentrations of PVA into PLGA 75:25 nanoparticles manufactured through microfluidics and showed that increasing PVA concentration resulted in increased nanoparticle size from approximately 200 to 500 nm and lower PDIs. The measured zeta potential was also reduced upon incorporation of PVA [10]. Kumar et al. also investigated the loading of insulin in PLGA 50:50 and 75:25 nanoparticles and demonstrated that addition of stabilisers into the formulation increased the particle size [28]. In contrast, other reported work using the water-in-oil-in-water (w/o/w) emulsion for the production of nanoparticles showed that increasing surfactant concentration produces smaller particles and the lack of stabiliser increases the particle size [29]. This may be due to the emulsifier being added to stabilise the emulsion during the formation of the nanoparticles. However, in the case of microfluidics, the nanoparticles are formed rapidly due to nanoprecipitation, thereby circumventing the need for additional stabilisers such as PVA. Indeed, such stabilisers may undermine the nanoprecipitation process and Chiesa et al. hypothesised that an increased viscosity due to incorporation of PVA into the microfluidics systems might decrease the mixing speed and, therefore, favour the formation of larger particles [10].

### Scale-independent manufacturing of nanoparticles: yield, purification and AT-line particle size monitoring

**Fig. 3:**
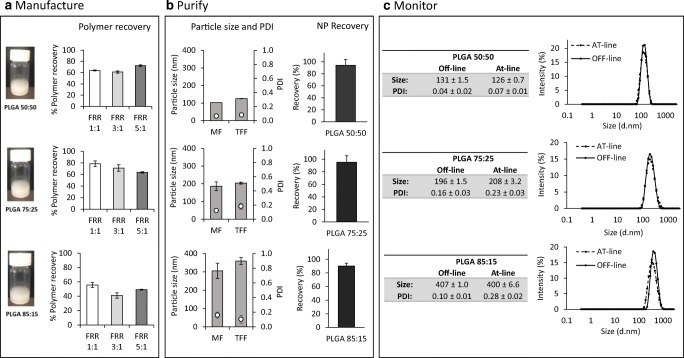
An overview of the process parameters measured during the production process of PLGA nanoparticles. **a** Manufacture—polymer recovery after manufacture of the PLGA 50:50, 75:25 and 85:15 nanoparticles formulated using microfluidics (MF) at TFR 10 mL/min and FRR 1:1, 3:1 and 5:1. **b** Purification via TFF—physicochemical characteristics before (MF) and after purification (TFF) and recovery of the polymeric nanoparticles (FRR 1:1, TFR 10 mL/min). **c** Monitoring—the particle size was monitored with Malvern OFF-line and AT-line in order to demonstrate the capability of the microfluidics method for continuous manufacturing of PLGA nanoparticles (particle sizes and intensity graphs for PLGA nanoparticles produced at FRR 1:1 and TFR 10 mL/min). For this study, nanoparticles without loaded protein were studied

The manufacture of nanoparticles should be scalable in order to succeed as a product for commercial use, and continuous and/or scale-independent processing offers several advantages over batch processing. For example, microfluidics, which is considered a scale-independent method for the manufacture of nanoparticles, can produce scalable volumes as determined by the run time. In contrast, batch processing is restricted by the size of the instrument used for the manufacture. For the purification of nanoparticles dialysis, centrifugation or gel filtration are commonly used within the laboratory setting; however, these methods are time consuming and difficult to scale up. Thus, cross flow filtration or tangential flow filtration (TFF) can be used for the removal of non-entrapped drug/protein from the nanoparticle suspensions. Therefore, to consider a manufacturing process which incorporates TFF purification and particle size monitoring, we assessed polymer recovery, particle size attributes and nanoparticle recovery after manufacture and TFF purification using nanoparticles prepared from PLGA 50:50, 75:25 and 85:15 without the addition of protein (Fig. 3).

With all three polymers, recovery was between 40 and 80% depending on the polymer and FRR used (Fig. 3a). The recovery of the PLGA 85:15 was significantly (*p* < 0.05) lower compared to the other PLGAs; the polymer content for 85:15 PLGA nanoparticles prepared at FRR 1:1 was 56 ± 4% whereas for FRR 3:1 and 5:1 the polymer content was 41 ± 4% and 49 ± 1% respectively (Fig. 3a). PLGA nanoparticles of 75:25 monomer ratio showed 79 ± 5%, 71 ± 6% and 63 ± 1% recovery for formulations prepared at FRR 1:1, 3:1 and 5:1 respectively (Fig. 3a). Regarding PLGA 50:50 formulations, the recovery was up to 64 ± 1%, 72 ± 5% and 72 ± 4% at FRR 1:1, 3:1 and 5:1, respectively. The high PLA ratio may be related to higher hydrophobicity and polymer precipitation within the cartridge [2]. Despite the lower polymer recovered observed compared to the high (˜ 100%) lipid recovery obtained after liposome manufacture using microfluidics showed previously [30], the values are in-line with those reported in the literature. Values between 40 and 80% are commonly reported when other techniques such as the double emulsion method (w/o/w), spray-drying or nanoprecipitation have been applied for the manufacture of polymers [31, 32]. In terms of particle attributes after purification, measurement of the particle size and size distribution after TFF demonstrated that neither the size of the particles, nor the PDI significantly changed after purification for all three PLGA nanoparticle formulations tested (Fig. 3b). Nanoparticle yield after purification was also high; all three copolymers showed a recovery over 90% (Fig. 3b).

The development of analytical methods which provide better information and control of the manufacturing process is required in particular in the case of nanomedicines, where size is a key critical quality attribute. The size of the particulate delivery systems can be measured by a wide range of techniques such as DLS, microscopy and particle tracking. Many of these techniques are only available for off-line measurements and cannot be applied for continuous processing. Real-time particle size analysis can be performed during continuous processing thus, any problem arising during production can be detected and corrected. The results in Fig. 3c show the addition of at-line particle size monitoring to the manufacturing process. The PLGA nanoparticles formulated using microfluidics at a selected TFR 10 mL/min and FRR 1:1 were sized using both an off-line system and an at-line system (real-time measurement). Results show the possibility to combine microfluidic fabrication, TFF purification and at-line particle size monitoring for PLGA nanoparticles.

### The effect of the microfluidic process parameters on the physicochemical characteristics and loading efficiencies of the PLGA nanoparticles

Within our work, we are interested in the development of PLGA nanoparticles as vaccine adjuvants therefore the manufacture of PLGA nanoparticles encapsulating a model antigen (OVA) was carried out using microfluidics. OVA is a well characterised and established protein with a molecular weight of ~ 45 kDa. Therefore, for the optimisation of the microfluidic parameters and the study of their influence in the protein loading, OVA was loaded in-line in a single step process by adding it in the aqueous phase. PLGA nanoparticles were prepared by altering the process parameters of the microfluidic system.

#### The effect of the process parameters on protein-loaded PLGA nanoparticles

**Fig. 4:**
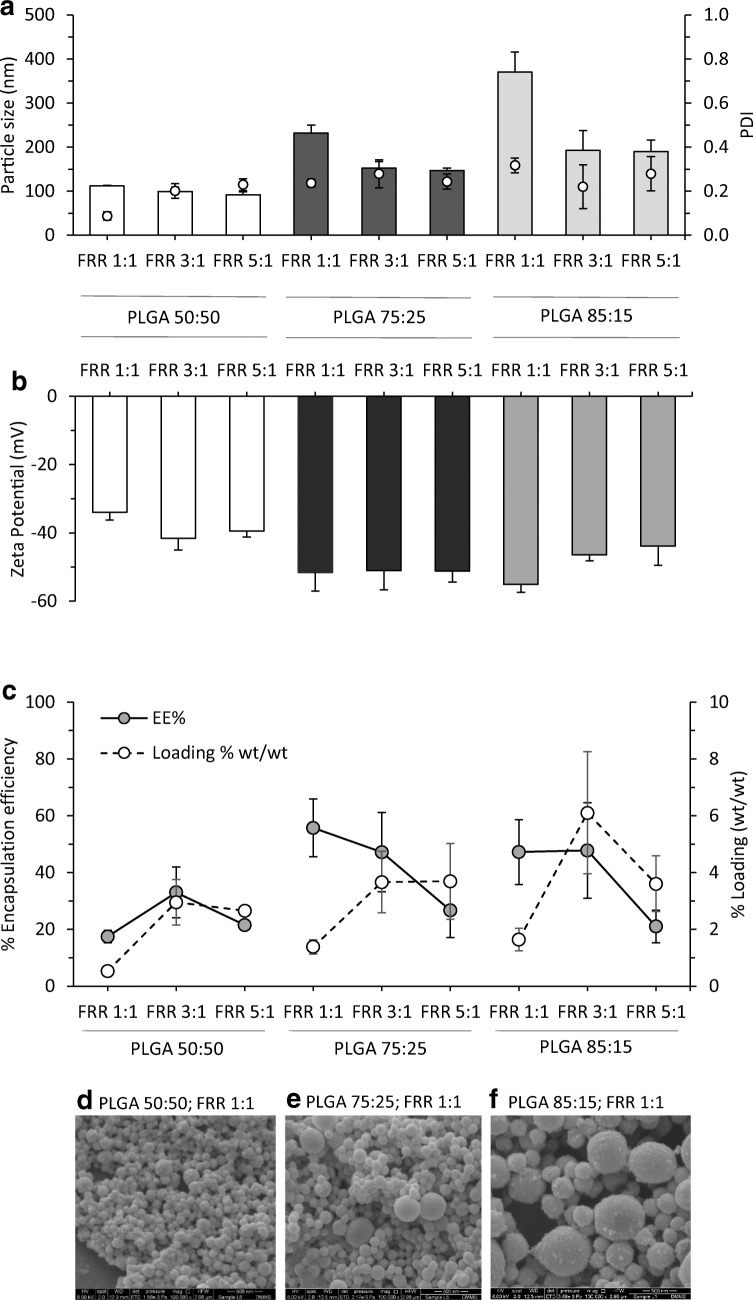
Incorporation of proteins within the PLGA nanoparticles. The effect of the FRR on the physicochemical characteristics of the PLGA nanoparticles incorporating OVA (TFR 10 mL/min): **a** particle size and particle size distribution, **b** zeta potential and **c** encapsulation efficiency (% of calculated concentration divided by initial amount added and multiplied by FRR dilution factor; 0.2 mg/mL OVA initial concentration) vs loading (wt/wt%). Results represent mean ± SD of at least triplicate measurements. SEM micrographs of OVA-loaded **d** PLGA 50:50, **e** PLGA 75:25 and **f** PLGA 85:15 nanoparticles formulated at FRR 1:1 and TFR 10 mL/min after purification

To develop protocols for incorporation of protein within the nanoparticles, the effect of the FRRs (1:1, 3:1 and 5:1) was evaluated on the PLGA 85:15, 75:25 and 50:50 copolymers loaded with a fixed initial amount of OVA and prepared at a fixed TFR of 10 mL/min (Fig. 4). Figure 4 a shows the particle size and PDI of the nanoparticles incorporating protein, which follows the same trend observed for the empty counterparts (Fig. 1); increasing FRR resulted in a decreased particle size, whereas this parameter did not affect the PDI. The copolymer 50:50 showed smallest sizes (below 115 nm) followed by copolymers 75:25 and 85:15. All the PDIs were below 0.3 in all the cases. However, the addition of 0.2 mg/mL protein increased (up to 30%) the size of some PLGA nanoparticles when compared to the ‘empty’ ones (Fig. 1 vs Fig. 4). In terms of their zeta potential, all three copolymers resulted in highly anionic nanoparticles as expected from PLGA-based systems with values from − 34 to − 55 mV (Fig. 4b) and no significant differences were observed between empty and loaded nanoparticles.

Figure 4 c shows the impact of the FRR on the percentage encapsulation efficiency (EE%) and loading capacity (% wt/wt) of PLGA nanoparticles at 0.2 mg/mL initial protein concentration. In general, increasing the FRR reduces the EE% with the FRR 5:1 tending to show the lowest antigen encapsulation with approximately 20–30% encapsulation of the initial amount loaded irrespective of the polymer used (Fig. 4c). This may be explained by the lower polymer concentration within the formulation as the initial polymer concentration was fixed at 10 mg/mL; therefore, when PLGA nanoparticles are produced at FRR 1:1, 50% of the initial amount will be in the final formulation. In the case of FRR 3:1 and FRR 5:1, the polymer content is reduced down to 25% and 16.7% respectively in the final suspension. To consider this, the results were also plotted as % wt/wt. When the ratio of protein loaded in relation to nanoparticle weight is considered, the loading tends to be lower at FRR 1:1 for all three polymers compared to 3:1 and 5:1 which gives similar loading (Fig. 4c). With all 3 polymers, the protein-loaded nanoparticles were spherical in nature and the particle size is seen to increase with increasing PLA:PGA ratio (Fig. 4d–f).

The influence of the copolymer ratio has been reported by Cao et al. [33]; when comparing PLGA 50:50 and PLGA 85:15 microparticles encapsulating OVA, PLGA 85:15 encapsulated approximately 24%, whereas the copolymer 50:50 only encapsulated ~ 15% [33]. Mukherjee et al. also showed that the higher hydrophobicity of PLGA 85:15 resulted in high protein loading compared to particles produced using PLGA 50:50 (38% vs 10% respectively) [34]. Similarly, De Rosa et al. produced PLGA microparticles using copolymers 50:50 and 75:25 and, again, the higher hydrophobicity and viscosity of the PLGA 75:25 resulted in higher EE% compared to PLGA 50:50 microparticles [35]. In terms of the microfluidic production, the use of PLGA with a higher hydrophobic content may promote more rapid aggregation and nanoparticle formation and thus capturing higher amounts of proteins. This may also explain why when high solvent levels are used; protein loading is lower as the higher solvent concentration will slow down nanoparticle formation.

**Fig. 5:**
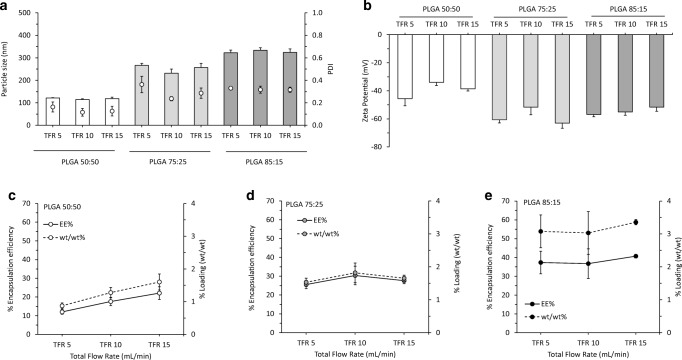
The effect of the TFR on the physicochemical characteristics of OVA loaded PLGA nanoparticles (FRR 1:1): **a** particle size (bars) and particle size distributions (dots) and **b** zeta potential. Encapsulation efficiency (EE%) (solid line) vs loading (wt/wt%) (dotted line) for **c** PLGA 50:50, **d** PLGA 75:25 and **e** PLGA 85:15. Results represent mean ± SD of triplicate measurements

After testing the FRR, the next step was to investigate the effect of the TFR in the formulation of these antigen loaded PLGA nanoparticles. In this case, the FRR was fixed at 1:1, whereas the TFR varied from 5 to 15 mL/min (Fig. 5). In terms of size, PDI and zeta potential, values were similar to those of ‘empty’ nanoparticles, with TFR having neither notable effect on size and PDI (Fig. 5a) nor zeta potential (Fig. 5b). Regarding protein encapsulation and loading capacity (Fig. 5c–e), again TFR made no notable impact. These results demonstrate higher processing speeds can be used for the production of polymeric PLGA nanoparticles and that the FRR and the polymer selection are the two key parameters dictating protein loading.

### The effect of the initial antigen concentration loaded in the physicochemical characteristics and encapsulation efficiency of the PLGA nanoparticles

**Fig. 6:**
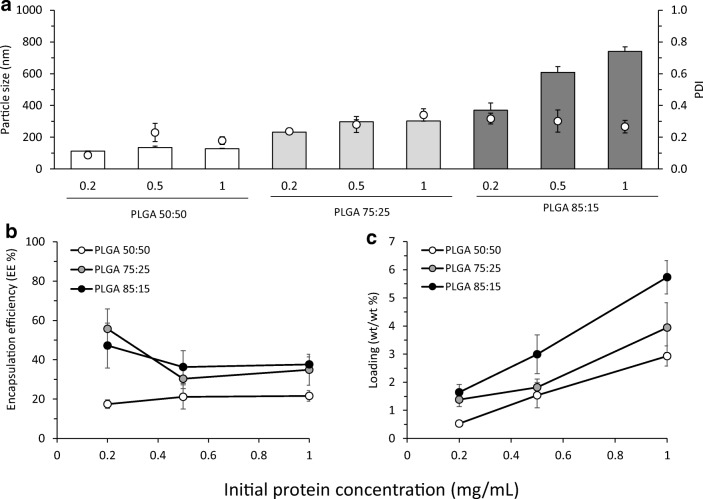
The effect of the initial protein concentration loaded on the physicochemical characteristics of the PLGA nanoparticles produced using microfluidics at FRR 1:1 and TFR 10 mL/min: **a** particle size (bars) and PDI (dots), **b** encapsulation efficiency (EE%) and **c** loading (wt/wt%) for PLGA 50:50, PLGA 75:25 and PLGA 85:15. Results represent mean ± SD of triplicate measurements

To consider the impact of varying OVA dose, different initial OVA concentrations (0.2, 0.5 and 1 mg/mL) were loaded in-line with the microfluidics system at a selected TFR of 10 mL/min and FRR 1:1 (0.1, 0.25 and 0.5 mg/mL OVA final concentrations after microfluidics respectively). Physicochemical characteristics of the formulations are shown in Fig. 6. The size of the particles notably increased for PLGA 85:15 nanoparticles from 300 to 800 nm (Fig. 6a). In contrast, PLGA 75:25 nanoparticles showed only small changes in size (from 240 to 300 nm), and PLGA 50:50 nanoparticles remained approximately the same (112 to 130 nm). All the nanoparticles were highly anionic, and no significant differences were found in terms of their zeta potential (results not shown).

In terms of varying the initial antigen concentration, the dominant factor in EE% is the polymer selection (Fig. 6b, c); EE% in PLGA 50:50 nanoparticles was approximately 20% irrespective of the initial protein concentration tested whilst PLGA 75:25 and 85:15 showed higher EE% (approx. 40–60%; Fig. 6b), suggesting that the nanoparticles have a wide range of entrapment capacity. In terms of % wt/wt loading, increasing the initial protein loading translated into high total loading within the particles and higher loading may be achievable but this may be at the detriment to particle size if more hydrophobic polymers are used (Fig. 6).

Previous studies have shown the change in the physicochemical characteristics of adjuvants systems upon incorporation of proteins [36]. In general, encapsulation of proteins within the nanoparticles provokes an increase in particle size. For example, Kissel et al. investigated the loading of BSA onto block polymers at different concentrations and showed that increasing BSA concentration produced an increased in the polymer microparticle size [37]. Yet, other studies have shown that incorporation of protein does not affect the physicochemical characteristics of the particles. For example, Azizi et al. produced BSA loaded PLGA 50:50 nanoparticles using different BSA concentrations (from 0.01 to 1% w/v) and the particle size of the nanoparticles did not change upon increasing the protein concentration [38].

### Varying the protein incorporated within the nanoparticles

To consider the impact of protein choice on nanoparticle formulation using microfluidics, different proteins were loaded at TFR of 10 mL/min and FRR 1:1. The selected antigens were as follows: BSA due to its larger molecular weight (Mw 66.5 kDa) and the TB vaccine candidate H56 (Mw 48.3 kDa). Protein loading was measured via HPLC. Table 1 outlines the physicochemical characteristics of the PLGA copolymers encapsulating these proteins at an initial protein concentration of 0.5 mg/mL (final concentration 0.25 mg/mL when produced at FRR 1:1). The results in Table 1 demonstrate that the type of protein loaded impacts on both the EE% and the physicochemical characteristics of the nanoparticles. With the PLGA 50:50 nanoparticles, the loading of the different proteins had no notable effect on particle size with particles being 135–150 nm in size (Table 1). Therefore, both the polymer adopted in the formulation and the protein being incorporated have to be considered as both impact on the attributes of the nanoparticles. Overall, the body of literature confirms that the use of PLGA of higher hydrophobic content promotes higher protein loading, which we hypothesise, might be the result of the hydrophobic polymers rapidly aggregating to form nanoparticles and thus capturing higher amounts of proteins. Indeed similar results have been shown by Koppolu et al. when loading different molecular weight proteins (insulin, OVA, BSA and concanavalin A) into chitosan based delivery systems [39]. The smallest molecular weight (insulin 6 kDa) resulted in near 100% EE whereas 66.5 kDa BSA resulted in 56% EE. OVA and H56 have similar molecular weights, and therefore, no significant differences were observed in their loading.

**Table 1:** Effect of the type of protein loaded on the physicochemical characteristics (particle size, PDI and zeta potential (ZP)) of the PLGA nanoparticles produced using microfluidics at FRR 1:1 and TFR 10 mL/min. Encapsulation efficiency was calculated as percentage of the calculated concentration divided by the theoretical concentration and loading capacity was calculated as the percentage of the mass of the loaded protein divided by the mass of the whole nanoparticle. Results represent mean ± SD of triplicate measurements

	Particle size (nm) ± SD	PDI ± SD	ZP (mV) ± SD	EE (%) ± SD	% wt/wt ± SD
Ovalbumin (OVA)					
PLGA 50:50	135 ± 9	0.23 ± 0.06	− 32 ± 5	21 ± 6	1.5 ± 0.4
PLGA 75:25	297 ± 12	0.28 ± 0.05	− 51 ± 3	35 ± 1	2.1 ± 0.1
PLGA 85:15	608 ± 36	0.3 ± 0.07	− 47 ± 2	36 ± 6	3.0 ± 0.5
Bovine serum albumin (BSA)					
PLGA 50:50	138 ± 6	0.11 ± 0.02	− 38 ± 2	25 ± 4	1.8 ± 0.3
PLGA 75:25	239 ± 9	0.26 ± 0.02	− 54 ± 2	31 ± 7	1.8 ± 0.4
PLGA 85:15	613 ± 72	0.35 ± 0.06	− 46 ± 2	23 ± 2	1.9 ± 0.2
Hybrid 56 (H56)					
PLGA 50:50	151 ± 6	0.24 ± 0.02	− 33 ± 2	7 ± 0.3	0.5 ± 0.02
PLGA 75:25	178 ± 3	0.13 ± 0.02	− 48 ± 2	35 ± 5	2.1 ± 0.3
PLGA 85:15	270 ± 11	0.16 ± 0.01	− 44 ± 2	38 ± 2	3.1 ± 0.2

## Conclusions

Through these studies, we have identified the critical process parameters in the microfluidic production of PLGA nanoparticles and outlined suggested process parameters that can be adopted for their scale-independent microfluidic manufacturing. By this method, proteins/antigens or peptides can be loaded in a single step into polymeric nanoparticles. Furthermore, the use of surfactants was deemed unnecessary using this production method. The formulation screening generated in this study can be applied to the encapsulation of proteins and antigens into PLGA nanoparticles, thus giving a scalable production pathway from laboratory tools to patient therapies.

## Data Availability

Data presented in this publication can be found at DOI: 10.15129/88155e7c-764b-405b-8f5f-2ebaf4017bb6.
